# Supplementary figures and images for: Single-protein detection in crowded molecular environments in cryo-EM images
Source: eLife. 2017 May 3;6:e25648. doi: 10.7554/eLife.25648 (PMC5453696; doi:10.7554/eLife.25648)

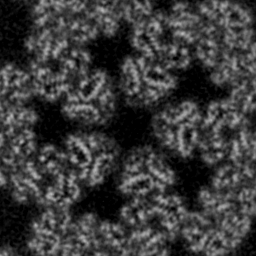

Supplement: Supplementary file 1. — Stack of sections from a reconstruction of the rotavirus RNA polymerase VP1 bound near a fivefold vertex in a double-layered particle (DLP; odd frames), interleaved with sections through a simulated DLP based on the model used for VP1 template generation (even frames). Voxel size is 0.1023 nm. DOI: http://dx.doi.org/10.7554/eLife.25648.014 [file elife-25648-supp1.tif]
