# Supplementary material for: Single-protein detection in crowded molecular environments in cryo-EM images
Source: eLife. 2017 May 3;6:e25648. doi: 10.7554/eLife.25648 (PMC5453696; doi:10.7554/eLife.25648)
Supplement: Supplementary file 5. — Columns Δf1, Δf2, and αast provide defocus parameters (Rohou and Grigorieff, 2015) assumed in template generation. DOI: http://dx.doi.org/10.7554/eLife.25648.018 [file elife-25648-supp5.docx]

| **File #** | **Image type, expected proteins** | **Structure** | | **Assumed defocus** | | |  |
| --- | --- | --- | --- | --- | --- | --- | --- |
|  |  | **Target protein** | **Source (PDB code)** | **Δ*f*_1_ (nm)** | **Δ*f*_2_ (nm)** | ***α_ast_* (rad.)** | **Referring fig(s).** |
| 01 | Experimental, apoferritin | Apoferritin | 2W0O | 216.3 | 224.9 | 0.34 | 1b,d,e |
| 02 |  | GroEL | 1GRL | 216.3 | 224.9 | 0.34 | 1c,d,e |
| 03 |  | Apoferritin (200 kDa fragment) | 2W0O | 216.3 | 224.9 | 0.34 | 2b; 2 - FS1 |
| 04 |  | Apoferritin (150 kDa fragment) |  | 216.3 | 224.9 | 0.34 | 2b; 2 - FS1 |
| 05 |  | Apoferritin (100 kDa fragment) |  | 216.3 | 224.9 | 0.34 | 2b; 2 - FS1 |
| 06 | Simulated, apoferritin | Apoferritin | 2W0O, 4F5S | 70.0 | 70.0 | 0.00 | 3a |
| 07 | Simulated, apoferritin + BSA |  |  | 70.0 | 70.0 | 0.00 | 3a |
| 08 |  |  |  | 70.0 | 70.0 | 0.00 | 4a |
| 09 |  |  |  | 2000.0 | 2000.0 | 0.00 | 3a; 3 - FS1 |
| 10 |  |  |  | PPM | | | 4a; 3 - FS1 |
| 11 |  |  |  | 70.0 | 70.0 | 0.00 | text |
| 12 |  |  |  | 2000.0 | 2000.0 | 0.00 | text |
| 13 |  |  |  | 70.0 | 70.0 | 0.00 | 5c,d |
| 14 |  |  |  | 230.0 | 230.0 | 0.00 | 5c,d |
| 15 |  |  |  | 1000.0 | 1000.0 | 0.00 | 5c,d |
| 16 |  |  |  | 2000.0 | 2000.0 | 0.00 | 5c,d |
| 17 |  |  |  | 2000.0 | 2000.0 | 0.00 | 5c,d |
| 18 |  |  |  | PPM | PPM | PPM | 5 - FS1 |
| 19 | Experimental, rotavirus DLP | Asymmetric subunit | 3KZ4 | 270.0 | 268.4 | -1.15 | 5b,c |
| 20 |  |  |  | 270.0 | 268.4 | -1.15 | 5c |
| 21 |  |  |  | 288.1 | 310.8 | 0.31 | 5c |
| 22 |  |  |  | 288.1 | 310.8 | 0.31 | 5c |
| 23 |  |  |  | 288.1 | 310.8 | 0.31 | 5c |
| 24 |  |  |  | 1463.8 | 1419.5 | -0.11 | 5c |
| 25 |  |  |  | 1463.8 | 1419.5 | -0.11 | 5c |
| 26 |  |  |  | 1591.4 | 1587.2 | -0.48 | 5c |
| 27 |  |  |  | 1591.4 | 1587.2 | -0.48 | 5c |
| 28 |  |  |  | 1591.4 | 1587.2 | -0.48 | 5b,c |

**Supplementary File 5.** List of files included in Supplementary File 4. Columns *Δf1*, *Δf2*, and *αast* provide defocus parameters (Rohou, A. & Grigorieff, N., J. Struct. Biol. 192(2):216-221, 2015) assumed in template generation.
